# The use of medicinal plants by the population from the Protected Landscape of “Serra de Montejunto”, Portugal

**DOI:** 10.1186/s13002-019-0309-0

**Published:** 2019-07-01

**Authors:** Cidália Vinagre, Sandra Vinagre, Ermelinda Carrilho

**Affiliations:** 1 Agrupamento de Escolas Severim de Faria – Évora, Estrada das Alcáçovas, 7005-205 Évora, Portugal; 2 Departamento de Matemática, CIMA, ECT, Universidade de Évora, Rua Romão Ramalho, 59, 7000-671 Évora, Portugal

**Keywords:** Ethnobotany, Medicinal plants, Ethnopharmacology, Protected Landscape of “Serra de Montejunto”, Portugal

## Abstract

**Background:**

Traditional medicine has an important role in local communities, who use plants in the treatment of various diseases. The research of traditional uses of medicinal plants allows us to document and analyze ethnopharmacological practices. This paper reports on an ethnobotanical survey that was conducted in the Protected Landscape of the “Serra de Montejunto”, a Portuguese area in the west of the Iberian Peninsula, where these studies were nonexistent.

**Methods:**

The information was obtained through semi-structured ethnobotanical interviews with 78 informants, who were selected from several zones from the study area to have a representative of the entire landscape, during 2014. Local medicinal uses of plants were identified and grouped into 10 categories through data analysis, in quantitative indices such as the relative frequency citation (RFC), the cultural importance index (CI), and the informant consensus factor (*F*_IC_). These were used to evaluate the importance of medicinal plants to the locals.

**Results:**

In the fieldwork, we found 105 *taxa* used as medicinal plants which belong to 46 families, where *Rosaceae*, *Asteraceae*, *Fabaceae*, and *Lamiaceae* are the ones with more diversity. The plants were grouped into 10 categories, where the digestive category is the most cited, with 54 *taxa*, and the ophthalmological category is the less cited, with only one *taxon*. Leaves and aerial parts are the components most used. Infusion is the most reported form of preparation, along with the oral administration. Most plants referred in this study are still in use today; only 17 are no longer used at the present time because habits have changed. A catalog of medicinal plants was also drawn up.

**Conclusion:**

This work enabled us to explore once more our experiences and memories as well as the ancestral use of plants with the goal of expanding ethnopharmacological knowledge. The absence of ethnobotanical studies in this region led us to gather information about useful plants and their applications and benefits. This research helps in the conservation effort of the collective knowledge of medicinal plants for future generations. However, a detailed analysis by body system is still required.

## Background

Plants have been used since ancient times by humans. Several purposes have been served by them such as food, spices, medicine, ritual components. The knowledge of plants and their benefits have been accumulated and passed on through the generations, through writing or memory. While some knowledge has been lost other has endured to present days and is still in use.

The scientific discipline dedicated to the relationship between man and the use of plants is called ethnobotany [1]. The American botanist John W. Harshberger coined the term “ethnobotany” in 1985 to describe studies of “plants used by primitive and aboriginal people” and in his 1896 publication, *The purposes of ethno-botany*, [2], he suggested “ethnobotany” be a field which elucidates the “cultural position of the tribes who used the plants for food, shelter or clothing,” generally accepted as a starting point for this field as an academic discipline [3]. Therefore, ethnobotany deals directly with the interrelationship between people and plants, including all forms of perception and appropriation of plant resources [4].

The human being has always tried to find in the plants that nature so lavishly offers sustenance as well as healing for various diseases that afflicted the course of its existence [5].

Plants have always been the primary source of treatment humanity used for disease and injury. Initially, they were used empirically, selected and tested. The knowledge of their effects and toxicity was then passed on. Through this process and collective memory, many plants are still used in the traditional way. The use of plants in therapy remains, worldwide, an important means of combating diseases. Medicinal herbal products in developing countries account for 80% of drugs used [6]. The same authors state that since 2002 the World Health Organization has launched its first global strategy on traditional medicine.

Several studies on the use and effects of medicinal plants have been conducted throughout the world with a marked increase in the Iberian Peninsula. In Portugal research on ethnobotanical projects was initiated by the Portuguese Institute for Nature Conservation and Forestry. The 2000’s study commissioned was titled: “Aromatic and/or Medicinal Plants in the National Network of Protected Areas”. In that study, the Protected Landscape of “Serra de Montejunto” was not featured. This article however focuses solely on that region.

While Portugal is a Mediterranean region due to its edaphoclimatic conditions, the country has a high phytodiversity and inherent resources with a high potential for medical purposes [6]. For some authors, the plants from the Mediterranean region have real medicinal potential [7]. With this work, we intended to verify that in the region studied, there is a great biodiversity and a documented use of medicinal plants.

## Methods

### Study area

**Fig. 1:**
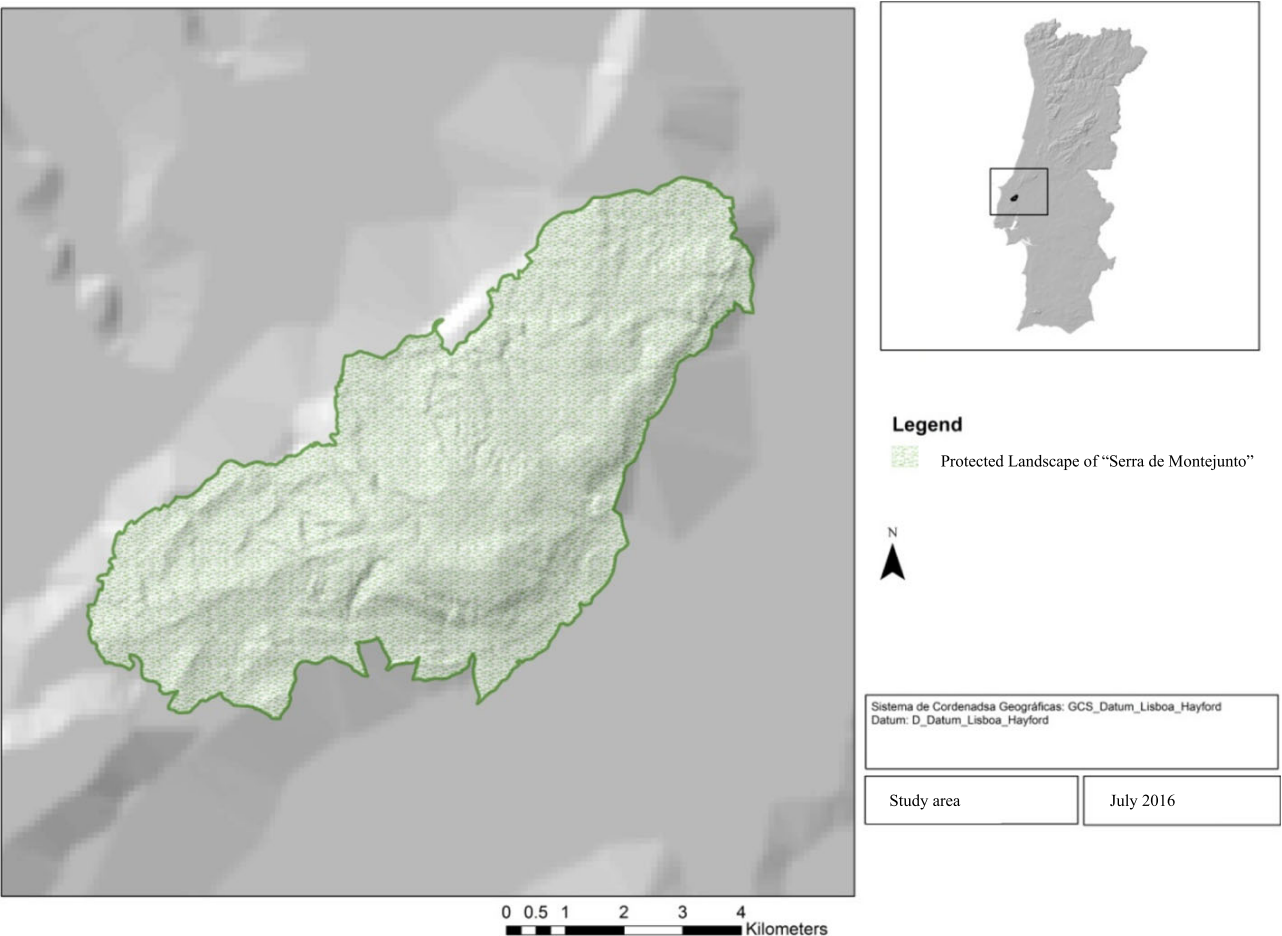
Location of the study area in Portugal

The Portuguese Protected Area of “Serra de Montejunto” was created in 1999 [8] due to the national importance of its natural vegetation. It is located in the western part of the Iberian Peninsula (Portugal), comprised of 4897.39 ha and stretches over 15 km with a northeast (NE)–southwest (SW) orientation; is limited by the Cadaval municipality (East) and the Alenquer municipality (West); and is in the Lisbon District (Fig. 1). It is part of the Dividing Portuguese Sector integrated into the inner Mediterranean Region [9, 10], and in a biogeographic context, it is a Mediterranean bioclimate, with a mesomediterranean thermotype and subhumid to humid ombrotype, according to the Rivas-Martinez Worldwide Bioclimatic Classification [11] and the Monteiro-Henriques maps [12].

This territory contains important biophysical characteristics resulting from its unique geography, in that the studied area, a large anticline of calcareous origin whose summit reaches an altitude of 666 m enjoys a privileged geographic location, between the coast and the Tagus Valley, encompassing the Montejunto and the Estrela mountainous, acting as a barrier to the oceanic influence, separating the rainiest part of the country from the driest. The Protected Landscape of the Montejunto Mountain, marked by millennia of anthropic action, still holds some vestiges of the primeval vegetation, which testify to the potential of the region’s plant life within the scope of the *Arisaro Simorrhini*-*Quercetum Broteroi* oak forest. Concerned to the flora of Mediterranean influence, more than 750 *taxa* have been identified, divided by 91 botanical families, of which the *Asteraceae*, *Fabaceae*, *Poaceae*, and *Lamiaceae* families have more than a third of the floristic variety [13].

This is the specific area this work was conducted in and refers to as it explores the use of medicinal plants by the locals.

### Data collection

The fieldwork was carried out in 2014, between March and December. Working with 78 informants, we obtained data about 105 medicinal *taxa*, belonging to 46 botanical families and a corresponding total of 2808 use-reports. The information obtained in the interviews was about wild or cultivated plants, which were obtained by the interviewees themselves or by their family, and purchased plants were never considered. Local medicinal uses were identified and grouped into 10 categories: circulatory, dermatological, digestive, neurological, ophthalmological, reproductive, respiratory, skeleton and muscles, urinary, and other uses.

In gathering the data for the project, we used semi-structured ethnobotanical interviews [1, 14] that, while informal, were acquiesced by the participants through oral agreement. The people interviewed, in their local language (Portuguese language), were either current or former residents of the area, selected because of their knowledge on the subject. Most were recommended by other locals when the topic came into question.

To complement the interviews and aid in the identification of the plants, the informants were presented with photograph portfolios and a herbarium created by the authors or invited to a field walk. Some were interviewed a second time in order to expand on the already compiled information.

This knowledge of ethnopharmacological plant uses was transmitted through oral traditions (parents or other relatives). Many of informants referred that this knowledge was also been complemented by personal experience (45) and the youngest informants reported other sources, such as books (22), television, radio, or internet (20).

Of the total 78 interviewees, 55 were women. That represents 70% of the sample. The age of the sample varied from 19 to 94 years, averaging at 68 years old. Around 50% of this group was already retired. While four had higher education, the majority was not scientific literate, either not having gone to school or not having more than primary education (Table 1).

**Table 1:** Demographic details of 78 informants

Category	Subcategory	Frequency
Gender	Male	23 (30%)
	Female	55 (70%)
Age	50 or less	12 (15.4%)
	51–60	8 (10.2%)
	61–70	24 (30.8%)
	71–80	21 (26.9%)
	81 or more	13 (16.7%)
Education level	Illiterate	21 (26.9%)
	Primary	29 (37.2%)
	Middle	15 (19.2%)
	Secondary	9 (11.6%)
	University	4 (5.1%)

### Botanical identification

All medicinal plants reported were identified using the following literature: Coutinho [15], Franco [16, 17], Franco and Rocha-Afonso [18–20], and Castroviejo et al. [21–36]. To compare the existing Portuguese local names, we used Rocha [37], Fernandes and Carvalho [38], and Arias [39], and the scientific names of plant species were confirmed in accordance with the International Index of Plant Name (http://www.ipni.org) and the Plant List database (http://www.theplantlist.org).

Voucher specimens were prepared and deposited in the Herbarium “João de Carvalho e Vasconcelos” of the “Instituto Superior de Agronomia” (LISI), University of Lisbon.

Most of these medicinal plants are part of the floristic inventory of the Protected Landscape of “Serra de Montejunto” [13].

### Ethnobotanical data analysis

This study was conducted in order to obtain data about the medicinal plants used in the region, their local Portuguese names, their medicinal uses and applications, preparation, administration, condition (fresh or dried), if it is actually used (yes or no), and parts of the plant used.

The information obtained during the interviews, recorded in Table 2, was statistically analyzed. The reported plants were grouped into 10 categories, based on the body systems, each of which is divided into several subcategories, in accordance with the information gathered from the informants.

**Table 2:** Plants with medicinal uses reported by at least three informants

Botanical family, scientific name	Local portuguese names	FC^a^	RFC^b^	Popular use	Part(s) used	Preparation	Administration	Condition	Actual use	UR_i_^c^	UR^d^	CI^e^	Voucher number
*Amaranthaceae*													
*Gomphrena globosa* L.	Perpétuas-roxas	4	0.05	Respiratory—hoarseness	Flower	Infusion	Oral	Dried	No	4	4	0.05	LISI 406/2019
*Amaryllidaceae*													
*Allium cepa* L.	Cebola	42	0.54	Respiratory—bronchitis, cough, hoarseness, throat	Onion skin	Infusion, syrup (sugar maceration, some with lemon or orange skin), vapors	Oral	Fresh or dried	Yes or no	44	51	0.65	LISI 357/2019
				Urinary—diuretic, urinary tract	Bulb	Direct ingestion	Oral	Fresh	Yes	3			
				Dermatological—furuncles	Bulb	Direct application	External	Fresh	Yes	2			
				Other—earache	Bulb	Direct application	External	Fresh	Yes	2			
*Allium sativum* L.	Alho, alho-comum	34	0.44	Digestive—constipation	Bulb	Direct ingestion	Oral	Fresh	Yes	2	46	0.59	LISI 358/2019
				Circulatory—blood pressure, blood purifier, cholesterol	Bulb	Direct ingestion	Oral	Fresh	Yes or no	8			
				Dermatological—cuts, furuncles, herpes, infection skin, insect bites, pimples, shingles, wounds, wounds on lips	Bulb	Direct application (some frying in olive oil or with oil from wheat)	External	Fresh	Yes or no	27			
				Skeleton and muscles—rheumatism	Bulb	Alcohol maceration (some with black bryony), direct ingestion	External, oral	Fresh	Yes or no	7			
				Other—earache	Bulb	Direct application (frying in olive oil)	External	Fresh	Yes	2			
*Apiaceae* (*Umbelliferae*)													
*Coriandrum sativum* L.	Coentros, coentro, coriandro	4	0.05	Urinary—diuretic	Aerial part	Direct ingestion	Oral	Fresh	Yes	4	4	0.05	LISI 377/2019
*Daucus carota* subsp. *sativus* (Hoffm.) Schübl. & G.Martens	Cenoura	71	0.91	Digestive—liver	Leaves	Infusion	Oral	Fresh	Yes	2	72	0.92	LISI 408/2019
				Respiratory—cold, cough	Root	Syrup (sugar maceration, some with juice of lemon or blue chalk sticks)	Oral	Fresh	Yes or no	70			
*Foeniculum vulgare* Mill.	Funcho, fiôlho	7	0.09	Digestive—digestion, intestines	Aerial part, fruit, seeds	Infusion	Oral	Fresh or dried	Yes or no	5	7	0.09	LISI 69/2019
				Urinary—diuretic	Aerial part	Infusion	Oral	Fresh or dried	Yes	2			
*Petroselinum crispum* (Mill.) A.W.Hill	Salsa	10	0.13	Digestive—digestion, disinfectant of the digestive system, stomach	Aerial part	Infusion	Oral	Fresh or dried	Yes	6	17	0.22	LISI 378/2019
				Urinary—diuretic	Aerial part	Direct ingestion	Oral	Fresh	Yes	4			
				Reproductive—gynecological infection, prostate	Aerial part	Direct ingestion, infusion	Oral	Fresh or dried	Yes	2			
				Other—anti-cancerous	Aerial part	Direct ingestion	Oral	Fresh	Yes	5			
*Asteraceae* (*Compositae*)													
*Arctium minus* Bernh.	Bardana, bardana-ordinária, pegamasso--menor	3	0.04	Digestive—liver	Root	Infusion	Oral	Fresh	Yes	3	6	0.08	LISI 379/2019
				Circulatory—blood purifier	Root	Infusion	Oral	Fresh	Yes	3			
*Chamaemelum nobile* (L.) All.	Macela, marcela, cabecinha-de-marcela, cabecinha-de-macela	19	0.24	Digestive—appetite, belly ache, digestion, parasites, stomach	Aerial part, flower	Cooking (with bran), infusion	External, oral	Fresh or dried	Yes or no	17	25	0.32	LISI 70/2019
				Urinary—diuretic	Aerial part, flower	Infusion	Oral	Fresh or dried	No	2			
				Neurological—tranquillizer	Flower	Infusion	Oral	Fresh or dried	Yes or no	2			
				Other—fever	Flower	Infusion	Oral	Fresh	No	4			
*Lactuca sativa* L.	Alface, alface-hortense	4	0.05	Neurological—tranquillizer	Leaves	Infusion	Oral	Fresh	Yes	4	4	0.05	LISI 359/2019
*Leucanthemum sylvaticum* (Brot.) Nyman	Margarida-branca, bem-me-quer, margarida--maior	3	0.04	Circulatory—blood pressure	Aerial part	Infusion	Oral	Fresh or dried	Yes	3	3	0.04	LISI 71/2019
*Matricaria recutita* L.	Camomila, margaça, margacinha, matricária	36	0.46	Digestive—digestion, stomach	Aerial part	Infusion	Oral	Fresh or dried	Yes	8	40	0.51	LISI 405/2019
				Neurological—tranquillizer	Aerial part, flower	Infusion	Oral	Fresh or dried	Yes or no	32			
*Senecio serpens* G.D.Rowley	Bálsamo	40	0.51	Respiratory—cough	Leaves	Syrup (sugar maceration, some with carrot)	Oral	Fresh	Yes	5	42	0.54	LISI 360/2019
				Dermatological—cicatrizing, insect bites, wounds	Leaves, sap	Direct application, ointment (with olive oil and elderberry)	External	Fresh	Yes or no	37			
*Silybum marianum* (L.) Gaertn.	Cardo-leiteiro, cardo-mariano, cardo-de--santa-maria	4	0.05	Circulatory—blood purifier	Aerial part	Infusion	Oral	Dried	Yes	4	4	0.05	LISI 356/2019
*Taraxacum officinale* F.H.Wigg.	Dente-de-leão, taráxaco	7	0.09	Digestive—intestines, liver	Leaves	Infusion	Oral	Fresh	Yes or no	6	10	0.13	LISI 120/2019
				Urinary—diuretic	Aerial part	Infusion	Oral	Dried	Yes	4			
*Boraginaceae*													
*Borago officinalis* L.	Borragem, erva-da-borragem, borago, chupa--mel	10	0.13	Respiratory—cold	Aerial part	Infusion	Oral	Fresh or dried	Yes	2	11	0.14	LISI 72/2019
				Dermatological—burns, infection skin, wounds	Leaves	Direct application (some heated in the candle), infusion	External	Fresh or dried	No	3			
				Other—fever	Aerial part, flower, leaves	Infusion	Oral	Fresh or dried	No	6			
*Brassicaceae (Cruciferae)*													
*Brassica napus* L.	Nabo, nabiça, colza	4	0.05	Respiratory—bronchitis, catarrh, cough, whooping cough	Root	Syrup	Oral	Fresh	Yes or no	8	8	0.10	LISI 361/2019
*Brassica oleracea* L.	Couve	4	0.05	Digestive—stomach, ulcers	Leaves	Direct ingestion (juice from leaves)	Oral	Fresh	No	4	4	0.05	LISI 362/2019
*Capsella bursa-pastoris* (L.) Medik.	Bolsa-de-pastor, erva-do-bom-pastor	12	0.15	Digestive—intestines, stomach	Aerial part, fruit	Infusion	Oral	Fresh or dried	No	4	20	0.26	LISI 73/2019
				Urinary—bladder, diuretic, kidney stone, urinary infection	Aerial part, fruit	Infusion	Oral	Fresh or dried	Yes or no	14			
				Circulatory—hemorrhages	Aerial part	Infusion	Oral	Fresh or dried	Yes	2			
*Cactaceae*													
*Opuntia maxima* Mill.	Figueira-da-índia, cato-dos-figos-da-índia, figueira-da-barbária	3	0.04	Respiratory—bronchitis, cough	Fruit, latex, leaves	Syrup	Oral	Fresh	No	3	3	0.04	LISI 380/2019
*Caprifoliaceae*													
*Sambucus nigra* L.	Sabugueiro, sabugo, sabugueiro-negro, sabugueiro-preto	16	0.21	Respiratory—influenza	Flower	Infusion	Oral	Fresh	Yes	2	17	0.22	LISI 74/2019
				Urinary—bladder, diuretic	Flower, leaves	Infusion	Oral	Fresh or dried	Yes or no	2			
				Dermatological—furuncles, hair, infection skin, wounds	Flower, leaves	Direct application (crushed leaves), infusion, ointment (some with olive oil and blue chalk sticks)	External	Fresh or dried	No	13			
*Caryophyllaceae*													
*Paronychia argentea* Lam.	Erva-prata, erva-dos-unheiros, erva-dos--linheiros, paroníquia	11	0.14	Digestive—stomach	Aerial part	Infusion	Oral	Fresh or dried	Yes or no	8	11	0.14	LISI 75/2019
				Circulatory—blood pressure	Aerial part	Infusion	Oral	Fresh or dried	Yes or no	3			
*Cistaceae*													
*Tuberaria lignosa* (Sweet) Samp.	Erva-da-desinfeção, erva-da-infeção, alcar, erva-das-túberas	16	0.21	Dermatological—wounds	Aerial part, leaves	Infusion	External	Fresh or dried	Yes or no	16	16	0.21	LISI 76/2019
*Cucurbitaceae*													
*Citrullus lanatus* (Thunb.) Matsum. & Nakai	Melancia, melancieira	4	0.05	Urinary—diuretic	Fruit	Direct ingestion	Oral	Fresh	Yes	4	4	0.05	LISI 412/2019
*Cucurbita maxima* Duchesne	Abóbora, aboboreira	5	0.06	Digestive—parasites, stomach	Fruit, seeds	Cooking (fruit pulp), direct ingestion	Oral	Fresh or dried	Yes	3	5	0.06	LISI 363/2019
				Reproductive—prostate	Seeds	Direct ingestion	Oral	Dried	Yes or no	2			
*Ecballium elaterium* (L.) A.Rich.	Pepino-de-são-gregório, pepineiro-de-são--gregório, pepineiro-bravo	6	0.08	Respiratory—sinusitis	Fruit	Direct application	External	Fresh	No	6	6	0.08	LISI 78/2019
*Cupressaceae*													
*Juniperus turbinata* Guss.	Zimbro	4	0.05	Urinary—diuretic	Fruit	Infusion	Oral	Dried	Yes	4	4	0.05	LISI 77/2019
*Dioscoreaceae*													
*Tamus communis* L.	Bódanha, baganha, norça-preta, uva-de-cão, arrebenta-boi	43	0.55	Skeleton and muscles—rheumatism	Fruit	Alcohol maceration (some with garlic)	External	Fresh	Yes or no	43	43	0.55	LISI 79/2019
*Equisetaceae*													
*Equisetum arvense* L.	Pinheirinha, cavalinha, cavalinha-dos--campos, erva-cavalinha, rabo-de-cavalo	40	0.51	Digestive—intestines, liver	Aerial part	Infusion	Oral	Fresh or dried	Yes	5	64	0.82	LISI 80/2019
				Urinary—bladder, diuretic, kidneys, urinary infection, urinary tract	Aerial part	Infusion	External, oral	Fresh or dried	Yes or no	36			
				Circulatory—blood pressure, blood purifier, cholesterol, diabetes, uric acid	Aerial part	Infusion	Oral	Fresh or dried	Yes or no	8			
				Skeleton and muscles—mineralizing	Aerial part	Infusion	Oral	Fresh or dried	Yes	3			
				Reproductive—gynecological infection, prostate	Aerial part	Infusion	External, oral	Fresh or dried	Yes or no	12			
*Equisetum telmateia* Ehrh.	Pinheirinha, cavalinha, rabo-de-cavalo	40	0.51	Digestive—intestines, liver	Aerial part	Infusion	Oral	Fresh or dried	Yes	5	64	0.82	LISI 81/2019
				Urinary—bladder, diuretic, kidneys, urinary infection, urinary tract	Aerial part	Infusion	External, oral	Fresh or dried	Yes or no	36			
				Circulatory—blood pressure, blood purifier, cholesterol, diabetes, uric acid	Aerial part	Infusion	Oral	Fresh or dried	Yes or no	8			
				Skeleton and muscles—mineralizing	Aerial part	Infusion	Oral	Fresh or dried	Yes	3			
				Reproductive—gynecological infection, prostate	Aerial part	Infusion	External, oral	Fresh or dried	Yes or no	12			
*Ericaceae*													
*Arbutus unedo* L.	Medronheiro, ervedeiro, êrvodo	4	0.05	Circulatory—cholesterol	Aerial part	Infusion	Oral	Fresh or dried	Yes	4	4	0.05	LISI 82/2019
*Calluna vulgaris* (L.) Hull.	Torga, urze, queiroga	5	0.06	Circulatory—gout	Leaves	Infusion	Oral	Fresh	No	5	5	0.06	LISI 83/2019
*Euphorbiaceae*													
*Euphorbia characias* L.	Leite-latrigueira, malateira-maior, trovisco-macho	23	0.29	Dermatological—warts	Latex	Direct application	External	Fresh	Yes	23	23	0.29	LISI 84/2019
*Fabaceae* (*Leguminosae*)													
*Cytisus grandiflorus* (Brot.) DC.	Giesta, giesta-das-sebes, giesteira-das-sebes	5	0.06	Digestive—liver	Flower	Infusion	Oral	Fresh or dried	No	3	8	0.10	LISI 85/2019
				Urinary—kidneys	Flower	Infusion	Oral	Fresh or dried	No	3			
				Circulatory—heart	Flower	Infusion	Oral	Fresh	No	2			
*Lupinus albus* L.	Tremoceiro, tremoceiro-branco, tremoço, tremoço-branco	5	0.06	Circulatory—cholesterol, diabetes	Seeds	Cooking, direct ingestion (water seeds maceration)	Oral	Dried	Yes or no	5	5	0.06	LISI 382/2019
*Phaseolus vulgaris* L.	Feijoeiro, feijoeiro-vulgar, feijão	3	0.04	Circulatory—diabetes	Pericarp	Infusion	Oral	Dried	Yes	3	3	0.04	LISI 383/2019
*Pterospartum tridentatum* (L.) Willk.	Carqueja, carqueija, carqueijeira	30	0.38	Digestive—diarrhea, liver, stomach	Aerial part, flower	Infusion	Oral	Fresh or dried	Yes or no	7	44	0.56	LISI 86/2019
				Respiratory—asthma, cold	Aerial part, flower	Infusion	Oral	Fresh or dried	Yes or no	2			
				Urinary—bladder, diuretic, kidneys	Flower, leaves	Infusion	Oral	Fresh or dried	Yes	6			
				Circulatory—blood pressure, blood purifier, cholesterol, diabetes, heart	Aerial part, flower, leaves	Infusion	Oral	Fresh or dried	Yes or no	24			
				Neurological—tranquillizer	Aerial part, flower	Infusion	Oral	Fresh or dried	Yes	3			
				Reproductive—prostate	Flower	Infusion	Oral	Dried	Yes	2			
*Ulex airensis* Esp.Santo, Cubas, Lousã, C.Pardo & J.C.Costa	Tojo	3	0.04	Digestive—liver	Flower	Infusion	Oral	Fresh or dried	No	3	3	0.04	LISI 87/2019
*Ulex jussiaei* Webb	Tojo, tojo-durázio	3	0.04	Digestive—liver	Flower	Infusion	Oral	Fresh or dried	No	3	3	0.04	LISI 88/2019
*Ulex minor* var. *lusitanicus* (Webb) C.Vicioso	Tojo, tojo-molar, tojo-branco, tojo-gatanho--menor	3	0.04	Digestive—liver	Flower	Infusion	Oral	Fresh or dried	No	3	3	0.04	LISI 89/2019
*Vicia faba* L.	Faveira, fava	4	0.05	Urinary—kidneys	Flower	Infusion	Oral	Dried	Yes	2	10	0.13	LISI 364/2019
				Circulatory—gout	Flower	Infusion	Oral	Dried	Yes	2			
				Dermatological—to stop bleeding, wounds	Seed coat	Direct application	External	Dried	No	4			
				Skeleton and muscles—rheumatism	Flower	Infusion	Oral	Dried	Yes	2			
*Fagaceae*													
*Quercus coccifera* L.	Carrasco, carrasqueiro	22	0.28	Digestive—diarrhea	Leaves	Infusion	Oral	Fresh or dried	Yes or no	18	25	0.32	LISI 90/2019
				Circulatory—blood purifier, cholesterol, diabetes	Leaves	Infusion	Oral	Fresh or dried	Yes	7			
*Gentianaceae*													
*Centaurium erythraea* Rafn	Fel-da-terra, centáurea-comum	14	0.18	Digestive—appetite, liver, parasites	Aerial part	Infusion	Oral	Fresh or dried	No	6	15	0.19	LISI 91/2019
				Circulatory—diabetes	Aerial part	Infusion	Oral	Fresh or dried	Yes or no	9			
*Geraniaceae*													
*Geranium purpureum* Vill.	Erva-de-são-roberto, erva-roberta	39	0.50	Digestive—digestion, gall bladder, intestines, liver, stomach	Aerial part	Infusion	Oral	Fresh or dried	Yes or no	39	58	0.74	LISI 92/2019
				Urinary—bladder, diuretic	Aerial part	Infusion	Oral	Fresh or dried	Yes or no	8			
				Circulatory—cholesterol, diabetes	Aerial part	Infusion	Oral	Fresh or dried	Yes	8			
				Other—anti-cancerous	Aerial part	Infusion	Oral	Fresh or dried	Yes or no	3			
*Hypericaceae*													
*Hypericum perforatum* L.	Hipericão, pelicão, plicão, milfurada, erva--de-são-joão, hipiricão-do-gerês, piricão	58	0.74	Digestive—digestion, hemorrhoids, liver, stomach, ulcers	Aerial part, flower, leaves	Infusion	Oral	Fresh or dried	Yes or no	70	76	0.97	LISI 93/2019
				Urinary—kidneys, urinary infection	Aerial part	Infusion	External, oral	Fresh or dried	Yes or no	3			
				Circulatory—blood pressure	Aerial part	Infusion	Oral	Fresh or dried	Yes	3			
*Juglandaceae*													
*Juglans regia* L.	Nogueira, nogueira-comum, nogueira--europeia, noz	28	0.36	Digestive—toothache	Leaves	Infusion	To rinse one’s mouth	Fresh or dried	Yes	3	38	0.49	LISI 384/2019
				Urinary—kidneys, urinary infection	Leaves	Infusion (some with mallows)	External, oral	Fresh or dried	Yes or no	5			
				Circulatory—chilblains, cholesterol, diabetes, heart	Leaves	Infusion	External, oral	Fresh or dried	Yes or no	7			
				Dermatological—hair loss, impetigo skin, wounds	Leaves	Infusion (some with mallows)	External	Fresh or dried	Yes or no	17			
				Reproductive—gynecological infection, prostate	Leaves	Infusion (some with mallows or leaves of orange tree)	External, irrigation	Fresh or dried	Yes or no	6			
*Lamiaceae* (*Labiatae*)													
*Melissa officinalis* L.	Erva-cidreira, melissa, limonete, chá-de--frança, citronela	71	0.91	Digestive–colic, digestion, intestines, stomach	Aerial part, leaves	Infusion	Oral	Fresh or dried	Yes or no	63	101	1.29	LISI 131/2019
				Urinary–diuretic, kidneys	Aerial part	Infusion	Oral	Fresh or dried	Yes	5			
				Neurological—headache, tranquillizer	Aerial part, leaves	Infusion	Oral	Fresh or dried	Yes or no	33			
*Mentha pulegium* L.	Poejo, poêjo, poejos, hortelã-pimenta-mansa	5	0.06	Respiratory—cough	Aerial part	Infusion	Oral	Fresh	No	5	5	0.06	LISI 94/2019
*Mentha spicata* L.	Hortelã, hortelã-comum, hortelã-verde-dos-açores	31	0.40	Digestive—digestion, intestines, parasites, stomach	Aerial part, leaves	Infusion	Oral	Fresh or dried	Yes or no	29	38	0.49	LISI 366/2019
				Respiratory—influenza	Aerial part	Infusion	Oral	Fresh	No	4			
				Neurological—tranquillizer	Aerial part	Infusion	Oral	Fresh	Yes	5			
*Mentha* x *piperita* L.	Hortelã-pimenta, hortelã-apimentada, hortelã	35	0.45	Digestive—digestion, flatulence, intestines, parasites, stomach	Aerial part, leaves	Infusion	Oral	Fresh or dried	Yes or no	40	45	0.58	LISI 365/2019
				Urinary—diuretic	Aerial part	Infusion	Oral	Fresh or dried	Yes	5			
*Origanum virens* Hoffmanns. & Link	Orégão, orégão-comum, oregãos, orégos, ourégão	5	0.06	Neurological—tranquillizer	Aerial part	Infusion	Oral	Fresh or dried	Yes	5	5	0.06	LISI 95/2019
*Prunella vulgaris* L.	Erva-férrea, prunela, brunela, consolda--menor	3	0.04	Dermatological—wounds	Leaves	Infusion	External	Fresh	No	3	3	0.04	LISI 96/2019
*Rosmarinus officinalis* L.	Alecrim, alecrim-da-terra, alecrinzeiro	15	0.19	Digestive—digestion, liver	Aerial part	Infusion	Oral	Fresh	Yes	3	18	0.23	LISI 97/2019
				Respiratory—bronchitis	Aerial part	Alcohol maceration (patches)	External	Fresh	No	2			
				Circulatory—cholesterol, to stimulate the circulation	Aerial part	Infusion	Oral	Fresh or dried	Yes	3			
				Neurological—tranquillizer	Aerial part	Infusion	Oral	Fresh or dried	Yes	4			
				Dermatological—to strengthen the hair	Aerial part	Infusion	External, oral	Fresh or dried	Yes or no	6			
*Thymus sylvestris* Hoffmans. & Link	Tomilho, sal-da-terra, serpão-do-monte	3	0.04	Respiratory—asthma, cold, cough	Aerial part	Infusion	Oral	Fresh or dried	Yes	5	5	0.06	LISI 98/2019
*Linaceae*													
*Linum usitatissimum* L.	Linho, linho-comum, sementes-de-linhaça	14	0.18	Digestive—constipation	Seeds	Direct ingestion	Oral	Dried	Yes	3	22	0.28	LISI 99/2019
				Respiratory—breathing difficulties, breathlessness, bronchitis, cold, cough, influenza, hoarseness	Seeds	Cooking, poultice	External	Fresh or dried	No	13			
				Dermatological—furuncles	Seeds	Poultice	External	Dried	No	2			
				Other—mumps	Seeds	Cooking	External	Dried	No	4			
*Malvaceae*													
*Lavatera cretica* L.	Malvas, malva, malva-bastarda, lavatera, lavatera-silvestre	73	0.94	Digestive—constipation, digestion, enteritis, hemorrhoids, infection of the mouth, intestines, oral hygiene, stomach	Aerial part, leaves	Infusion	Enema, external, oral, to rinse one’s mouth	Fresh or dried	Yes or no	36	168	2.15	LISI 385/2019
				Respiratory—throat	Leaves	Infusion	Gargle	Fresh	Yes or no	5			
				Urinary—urinary infection	Aerial part, leaves, seeds	Infusion (some with leaves of walnut tree), vapors	External, oral	Fresh or dried	Yes or no	50			
				Dermatological—infection skin, wounds	Aerial part, leaves	Infusion (some with leaves of walnut tree)	External	Fresh or dried	Yes or no	39			
				Reproductive—gynecological infection, intimate hygiene	Aerial part, leaves, seeds	Infusion (some with leaves of walnut tree or orange tree), vapors	Enema, external, irrigation	Fresh or dried	Yes or no	38			
*Malva hispanica* L.	Malvas, malva, malva-de-espanha	73	0.94	Digestive—constipation, digestion, enteritis, hemorrhoids, infection of the mouth, intestines, oral hygiene, stomach	Aerial part, leaves	Infusion	Enema, external, oral, to rinse one’s mouth	Fresh or dried	Yes or no	36	168	2.15	LISI 100/2019
				Respiratory—throat	Leaves	Infusion	Gargle	Fresh	Yes or no	5			
				Urinary—urinary infection	Aerial part, leaves, seeds	Infusion (some with leaves of walnut tree), vapors	External, oral	Fresh or dried	Yes or no	50			
				Dermatological—infection skin, wounds	Aerial part, leaves	Infusion (some with leaves of walnut tree)	External	Fresh or dried	Yes or no	39			
				Reproductive—gynecological infection, intimate hygiene	Aerial part, leaves, seeds	Infusion (some with leaves of walnut tree or orange tree), vapors	Enema, external, irrigation	Fresh or dried	Yes or no	38			
*Malva sylvestris* L.	Malvas, malva, malva-silvestre, malva--selvagem	73	0.94	Digestive—constipation, digestion, enteritis, hemorrhoids, infection of the mouth, intestines, oral hygiene, stomach	Aerial part, leaves	Infusion	Enema, external, oral, to rinse one’s mouth	Fresh or dried	Yes or no	36	168	2.15	LISI 404/2019
				Respiratory—throat	Leaves	Infusion	Gargle	Fresh	Yes or no	5			
				Urinary—urinary infection	Aerial part, leaves, seeds	Infusion (some with leaves of walnut tree), vapors	External, oral	Fresh or dried	Yes or no	50			
				Dermatological—infection skin, wounds	Aerial part, leaves	Infusion (some with leaves of walnut tree)	External	Fresh or dried	Yes or no	39			
				Reproductive—gynecological infection, intimate hygiene	Aerial part, leaves, seeds	Infusion (some with leaves of walnut tree or orange tree), vapors	Enema, external, irrigation	Fresh or dried	Yes or no	38			
*Moraceae*													
*Ficus carica* L.	Figueira, figueira-comum, bebereira	21	0.27	Respiratory—cold, cough	Fruit	Syrup (with dried apple and raisins)	Oral	Dried	No	4	24	0.31	LISI 381/2019
				Dermatological—callus, warts	Latex	Direct application	External	Fresh	Yes or no	20			
*Myrtaceae*													
*Eucalyptus globulus* Labill.	Eucalipto, eucalipto-comum	66	0.85	Respiratory—breathing difficulties, bronchitis, cold, cough, throat	Flower, leaves, young shoots	Infusion, syrup, vapors	Inhalation, oral	Fresh	Yes or no	75	77	0.99	LISI 101/2019
				Dermatological—wounds	Leaves	Infusion	External	Fresh	Yes or no	2			
*Myrtus communis* L.	Murta, murteira, murtinho, mirto	4	0.05	Dermatological—rash	Leaves	Direct application (powder from crushed leaves)	External	Dried	No	4	4	0.05	LISI 102/2019
*Oleaceae*													
*Fraxinus angustifolia* Vahl	Freixo, freixo-comum, freixo-de-folhas-estreitas	18	0.23	Digestive—constipation	Leaves	Infusion	Oral	Dried	Yes	2	32	0.41	LISI 103/2019
				Urinary—diuretic	Leaves	Infusion	Oral	Fresh or dried	Yes	4			
				Circulatory—blood pressure, blood purifier, cholesterol, gout, heart, uric acid	Leaves	Infusion	Oral	Fresh or dried	Yes or no	17			
				Skeleton and muscles—mineralizing, osteoporosis, rheumatism	Leaves	Infusion	Oral	Fresh or dried	Yes	9			
*Olea europaea* var. *europaea*	Oliveira	46	0.59	Circulatory—blood pressure, cholesterol, heart	Leaves	Infusion	Oral	Fresh or dried	Yes or no	48	54	0.69	LISI 368/2019
				Dermatological—furuncles, shingles, wounds	Olive oil	Direct application (frying garlic in olive oil or with oil from wheat), ointment (with blue chalk sticks and elderberry)	External	Fresh	Yes or no	4			
				Other—earache	Olive oil	Direct application (frying garlic in olive oil)	External	Fresh	Yes	2			
*Oxalidaceae*													
*Oxalis pes-caprae* L.	Erva-praga, erva-pata, erva-azeda-amarela, erva-canária, trevo-azedo	3	0.04	Digestive—parasites	Stem	Direct ingestion	Oral	Fresh	No	3	3	0.04	LISI 369/2019
*Papaveraceae*													
*Chelidonium majus* L.	Celidónia, erva-do-betadine, erva-andorinha, erva-das-verrugas	30	0.38	Circulatory—chilblains	Sap	Direct application	External	Fresh	Yes	3	34	0.44	LISI 104/2019
				Dermatological—cuts, wounds	Sap	Direct application	External	Fresh	Yes or no	31			
*Fumaria officinalis* L.	Cãezinhos, erva-moleirinha, fumária	3	0.04	Digestive—liver	Aerial part	Infusion	Oral	Dried	No	3	3	0.04	LISI 105/2019
*Phyllanthaceae*													
*Phyllanthus niruri* L.	Quebra-pedra, quebra-pedras, filanto	3	0.04	Urinary—kidney stone	Aerial part	Infusion	Oral	Fresh or dried	Yes	3	3	0.04	LISI 402/2019
*Pinaceae*													
*Pinus pinaster* Aiton	Pinheiro-bravo, pinheiro-marítimo	12	0.15	Circulatory—diabetes	Leaves	Infusion	Oral	Fresh	Yes or no	6	12	0.15	LISI 106/2019
				Dermatological—cicatrizing, wounds	Resin	Direct application	External	Fresh	No	6			
*Pinus pinea* L.	Pinheiro-manso	13	0.17	Respiratory—breathing difficulties, cough	Leaves, young shoots	Syrup (sugar maceration), vapors	Inhalation, oral	Fresh	Yes or no	12	14	0.18	LISI 107/2019
				Dermatological—cicatrizing, wounds	Resin	Direct application	External	Fresh	No	2			
*Plantaginaceae*													
*Plantago major* L.	Tanchagem, tanchagem-maior, erva-das-sete-linhas	3	0.04	Digestive—hemorrhoids	Leaves	Infusion	Oral	Fresh	Yes	2	6	0.08	LISI 108/2019
				Respiratory—cough	Leaves	Infusion	Oral	Fresh	Yes	2			
				Urinary—urinary infection	Leaves	Infusion	Oral	Fresh	Yes	2			
*Poaceae* (*Gramineae*)													
*Cymbopogon citratus* (DC.) Stapf	Chá-príncipe, erva-príncipe, erva-limão	31	0.40	Digestive—digestion, colic, liver, stomach	Aerial part, leaves	Infusion	Oral	Fresh or dried	Yes or no	25	46	0.59	LISI 386/2019
				Neurological—antidepressant, tranquillizer	Aerial part, leaves	Infusion	Oral	Fresh or dried	Yes	21			
*Hordeum vulgare* L.	Cevada-santa, cevada	3	0.04	Skeleton and muscles—rheumatism	Seeds	Poultice (with flour)	External	Dried	No	3	3	0.04	LISI 399/2019
*Triticum aestivum* L*.*	Trigo, trigo-mole	29	0.37	Digestive—belly ache, diarrhea	Seeds	Cooking (flour or bran with chamomile)	External, oral	Dried	Yes or no	10	34	0.44	LISI 400/2019
				Respiratory—bronchitis, cold, measles	Seeds	Cooking (bran), poultice (with flour and bran)	External	Dried	No	3			
				Dermatological—shingles	Seeds	Cooking, direct application (oil from seeds, some with garlic), poultice	External	Dried	No	21			
*Zea mays* L.	Milho, milho-grosso	64	0.82	Urinary—bladder, diuretic, urinary infection, urinary tract	Silk	Infusion	External, oral	Fresh or dried	Yes or no	71	78	1.00	LISI 387/2019
				Circulatory—blood pressure	Silk	Infusion	Oral	Dried	Yes	2			
				Skeleton and muscles—rheumatism	Corn kernel	Poultice (with flour)	External	Dried	No	2			
				Reproductive—gynecological infection, prostate	Silk	Infusion	External, oral	Dried	Yes or no	3			
*Polygonaceae*													
*Rumex conglomeratus* Murray	Labaça-ordinária, labaça, alabaça, regalo-da--horta	5	0.06	Digestive—diarrhea	Leaves	Infusion	Oral	Fresh	No	3	5	0.06	LISI 109/2019
				Dermatological—psoriasis	Root	Infusion	External	Fresh	Yes	2			
*Pteridaceae*													
*Adiantum capillus-veneris* L.	Avenca, avenca-das-fontes, capilária	6	0.08	Urinary—bladder, diuretic, kidneys, urinary tract	Aerial part	Infusion	Oral	Fresh or dried	Yes or no	6	6	0.08	LISI 110/2019
*Rosaceae*													
*Agrimonia eupatoria* L.	Agrimónia, erva-eupatória, erva-hepática	4	0.05	Digestive—digestion, liver, stomach	Aerial part	Infusion	Oral	Fresh or dried	Yes or no	5	5	0.06	LISI 111/2019
*Crataegus monogyna* Jacq.	Pilriteiro, espinheiro, pirliteiro	3	0.04	Circulatory—blood pressure, to stimulate the circulation	Flower, fruit, leaves	Infusion	Oral	Fresh or dried	Yes	5	5	0.06	LISI 112/2019
*Cydonia oblonga* Mill.	Marmeleiro, gamboeiro	6	0.08	Digestive—diarrhea	Leaves	Infusion	Oral	Fresh or dried	Yes	2	6	0.08	LISI 388/2019
				Circulatory—cholesterol, urea	Leaves	Infusion	Oral	Fresh or dried	Yes or no	4			
*Eriobotrya japonica* (Thunb.) Lindl.	Nespereira, nespereira-do-japão, nêsperas	22	0.28	Circulatory—blood pressure, cholesterol, diabetes	Leaves	Infusion	Oral	Fresh or dried	Yes or no	25	25	0.32	LISI 389/2019
*Fragaria vesca* L.	Morangueiro, morango, morangueiro-bravo, morangueira-vulgar, fragária, erva-dos-morangos	3	0.04	Digestive—diarrhea	Leaves	Infusion	Oral	Fresh	Yes	2	4	0.05	LISI 390/2019
				Urinary—kidneys	Leaves	Infusion	Oral	Fresh	Yes	2			
*Malus domestica* Borkh.	Macieira, maceira, maçãzeira	3	0.04	Respiratory—cold, cough	Fruit	Syrup (with dried figs and raisins)	Oral	Dried	No	4	4	0.05	LISI 410/2019
*Prunus avium* (L.) L.	Cerejeira, cerdeira, cerdeiro, cereja	53	0.68	Urinary—bladder, diuretic, kidneys, urinary infection, urinary tract	Fruit peduncles	Infusion	Oral	Fresh or dried	Yes or no	57	57	0.73	LISI 391/2019
*Prunus cerasus* L.	Ginjeira, ginjeiro, ginja	3	0.04	Urinary—bladder, kidneys, urinary tract	Fruit peduncles	Infusion	Oral	Fresh or dried	Yes or no	5	5	0.06	LISI 392/2019
*Prunus domestica* L.	Ameixeira, ameixieira, ameixoeira	6	0.08	Digestive—constipation, intestines	Fruit	Direct ingestion	Oral	Fresh or dried	Yes	6	6	0.08	LISI 393/2019
*Pyrus communis* L.	Pereira, pereira-mansa, pereira-comum	4	0.05	Digestive—digestion, gall bladder	Leaves	Infusion	Oral	Dried	Yes	6	6	0.08	LISI 394/2019
*Rosa canina* L.	Roseiras, roseira, rosas	36	0.46	Ophthalmological—eyes, inflammations	Flower, petals	Infusion	External	Fresh	Yes or no	36	36	0.46	LISI 113/2019
*Rubus ulmifolius* Schott	Silva, silvas, amoras-silvestres	4	0.05	Circulatory—diabetes	Leaves	Infusion	Oral	Fresh	Yes	4	4	0.05	LISI 114/2019
*Rubiaceae*													
*Galium aparine* L.	Pegamaço, amor-de-hortelão	5	0.06	Digestive—hepatitis, liver	Aerial part	Infusion	Oral	Fresh	Yes or no	3	5	0.06	LISI 115/2019
				Circulatory—blood purifier	Aerial part	Infusion	Oral	Fresh	Yes	2			
*Rutaceae*													
*Citrus limon* (L.) Osbeck	Limoeiro, limão	75	0.96	Digestive—digestion	Leaves, lemon skin	Infusion	Oral	Fresh	Yes	15	100	1.28	LISI 370/2019
				Respiratory—cold, cough, hoarseness, throat, voice	Fruit, juice, leaves, lemon skin	Direct ingestion (juice), infusion, syrup (sugar maceration, some with onion skin)	Oral	Fresh	Yes or no	74			
				Circulatory—blood purifier, cholesterol, uric acid	Fruit, juice, leaves	Direct ingestion (juice with water), infusion	Oral	Fresh	Yes	6			
				Neurological—tranquillizer	Leaves, lemon skin	Infusion	Oral	Fresh	Yes or no	2			
				Other—to slim	Fruit, juice	Direct ingestion (juice with water)	Oral	Fresh	Yes	3			
*Citrus sinensis* (L.) Osbeck	Laranjeira, laranjeira-doce, laranja-doce, laranja	47	0.60	Digestive—constipation, digestion, liver	Flower, leaves, mesocarp, orange skin	Direct ingestion, infusion	Oral	Fresh or dried	Yes or no	23	51	0.65	LISI 371/2019
				Respiratory—cold, throat	Flower, fruit, leaves, orange skin	Infusion, syrup (sugar maceration, some with onion skin)	Oral	Fresh or dried	Yes	7			
				Circulatory—heart	Flower, leaves	Infusion	Oral	Fresh or dried	Yes or no	5			
				Neurological—tranquillizer	Flower, leaves, orange skin	Infusion	Oral	Fresh or dried	Yes or no	14			
				Reproductive—gynecological infection	Leaves	Infusion (with mallows and/or leaves of walnut tree)	Irrigation	Fresh	No	2			
*Solanaceae*													
*Atropa belladonna* L.	Beladona, erva-moura-furiosa, erva--midriática	4	0.05	Skeleton and muscles—rheumatism	Fruit	Alcohol maceration	External	Fresh	No	4	4	0.05	LISI 401/2019
*Capsicum frutescens* L.	Piripiri, malagueta, pimenteiro-de-caiena	4	0.05	Digestive—hemorrhoids	Fruit	Direct ingestion	Oral	Fresh or dried	Yes or no	4	4	0.05	LISI 372/2019
*Hyoscyamus albus* L.	Meimendro, mimendro, meimendro-branco	25	0.32	Digestive—toothache	Seeds	Cooking, direct application (crushed leaves), smoke (burned seeds), vapors (boiled seeds)	External	Fresh or dried	No	16	28	0.36	LISI 403/2019
				Dermatological—furuncles, wounds	Leaves	Direct application, poultice	External	Fresh or dried	No	9			
				Other—earache	Seeds	Smoke (burned seeds)	External	Fresh	No	3			
*Lycopersicon esculentum* Mill.	Tomateiro, tomate	4	0.05	Urinary—diuretic	Fruit	Direct ingestion	Oral	Fresh	Yes	4	4	0.05	LISI 373/2019
*Physalis peruviana* L.	Fisális, alquequenge-amarelo, tomatinho-de--capuz	11	0.14	`Digestive—stomach	Fruit	Direct ingestion	Oral	Fresh	Yes	4	16	0.21	LISI 374/2019
				Circulatory—blood purifier, cholesterol, diabetes, uric acid	Fruit	Direct ingestion	Oral	Fresh	Yes	10			
				Other—aphrodisiac	Fruit	Direct ingestion	Oral	Fresh	Yes	2			
*Solanum melongena* L.	Beringela	4	0.05	Circulatory—cholesterol	Fruit	Direct ingestion (water maceration of fruit)	Oral	Fresh	Yes	4	4	0.05	LISI 375/2019
*Solanum tuberosum* L.	Batateira, semilheira, batata	26	0.33	Digestive—stomach	Tuber	Direct ingestion (juice)	Oral	Fresh	Yes	2	26	0.33	LISI 411/2019
				Neurological—headache	Tuber	Direct application	External	Fresh	Yes or no	15			
				Dermatological—insect bites	Tuber	Direct application	External	Fresh	Yes or no	9			
*Tiliaceae*													
*Tilia cordata* Mill.	Tília, tília-de-folhas-pequenas	64	0.82	Digestive—digestion	Aerial part	Infusion	Oral	Fresh or dried	Yes	2	69	0.88	LISI 395/2019
				Circulatory—circulation, heart	Aerial part, leaves	Infusion	Oral	Fresh or dried	Yes	4			
				Neurological—tranquillizer	Aerial part, flower, leaves	Infusion	Oral	Fresh or dried	Yes or no	63			
*Urticaceae*													
*Parietaria judaica* L.	Alfavaca-de-cobra, alfavaca, parietária, erva--das-paredes, erva-dos-muros	34	0.44	Digestive—hemorrhoids, infection of the mouth, intestines	Aerial part, leaves	Direct application (some crushed leaves and/or juice), infusion, vapors	External, oral, to rinse one’s mouth	Fresh or dried	Yes or no	11	61	0.78	LISI 116/2019
				Urinary—kidneys, urinary infection	Aerial part, leaves	Infusion, vapors	External, oral	Fresh or dried	Yes or no	24			
				Dermatological—skin, wounds	Aerial part, leaves	Infusion	External	Fresh	Yes or no	12			
				Reproductive—gynecological infection, prostate	Aerial part, leaves	Infusion, vapors	External, oral	Fresh or dried	Yes or no	14			
*Urtica dioica* L.	Urtiga-de-cauda, urtigas, urtiga	14	0.18	Urinary—diuretic	Aerial part	Cooking, infusion	Oral	Fresh	Yes	3	24	0.31	LISI 409/2019
				Circulatory—anemia, blood, blood purifier, circulation, diabetes, gout	Aerial part	Cooking, direct application, direct ingestion (juice), infusion	External, oral	Fresh or dried	Yes or no	12			
				Skeleton and muscles—rheumatism	Aerial part	Cooking, direct application	External, oral	Fresh	Yes or no	7			
				Other—anti-inflammatory	Aerial part	Cooking	Oral	Fresh	Yes	2			
*Valerianaceae*													
*Valeriana officinalis* L.	Valeriana, valeriana-das-boticas, erva-dos--gatos	3	0.04	Neurological—tranquillizer	Aerial part, leaves	Infusion	Oral	Fresh	No	3	3	0.04	LISI 407/2019
*Verbenaceae*													
*Aloysia citrodora* Paláu	Lúcia-lima, bela-luísa, doce-lima, limonete	61	0.78	Digestive—digestion, spasms, stomach	Aerial part, leaves	Infusion	Oral	Fresh or dried	Yes	31	75	0.96	LISI 376/2019
				Neurological—tranquillizer	Aerial part, leaves	Infusion	Oral	Fresh or dried	Yes or no	44			
*Vitaceae*													
*Vitis vinifera* subsp. *sylvestris* (C.C.Gmel.) Hegi	Videira, videira-europeia, vinha, parreira, cepa	3	0.04	Respiratory—cold, cough	Fruit	Syrup (with dried apple and dried figs)	Oral	Dried	No	4	4	0.05	LISI 127/2019
*Xanthorrhoeaceae*													
*Aloe vera* (L.) Burm.f.	Aloé-vera, aloé, aloé-dos-barbados, babosa	35	0.45	Dermatological—burns, cicatrizing, insect bites, pimples, skin allergy, wounds	Sap	Direct application	External	Fresh	Yes or no	35	45	0.58	LISI 396/2019
				Skeleton and muscles—rheumatism	Sap	Direct application	External	Fresh	Yes	4			
				Other—anti-cancerous	Leaves	Syrup	Oral	Fresh	Yes or no	6			

^a^The number of informants that referred the *taxon*

^b^RFC=FC/*N*, where *N* is the total number of informants

^c^Use-reports of the *taxon* by illness category

^d^Use-reports of the *taxon*

^e^CI=UR/*N*

To establish a deeper pharmacological knowledge of this region, the data was also assessed using quantitative analysis, namely ethnobotanical richness (R), relative frequency citation (RFC), cultural importance index (CI), and informant consensus factor (*F*_IC_).

The quantitative data obtained allowed for solid comparisons with other similar studies.

#### Ethnobotanical richness

The ethnobotanical richness (*R*) is the number of useful medicinal species [40]. The result obtained will be compared with that of other equivalent studies carried out in Portugal [41–43].

#### Relative frequency citation

The relative frequency citation (RFC) is given by RFC = FC/*N*, where FC is the total number of informants that referred to the *taxon* and *N* is the total number of informants. This index reveals the importance of each species [44].

#### Cultural importance index

The cultural index (CI) is given by CI = UR/*N*, where UR (use-reports) is the use recorded for every *taxa* and *N* is the total number of informants. This index was used to estimate the cultural significance of each species, in other words, to verify, in quantitative terms, to what extent each species is present in the local culture and in the memory of the inhabitants in the study [45].

#### Informant consensus factor

The informant consensus factor (*F*_IC_), testing homogeneity on the informant’s knowledge, is given by the ratio between the number of use-reports (*n*_ur_) minus the number of *taxa* used (*n*_t_) and the number of use-reports minus one, that is, *F*_IC_ = (*n*_ur_ − *n*_t_)/(*n*_ur_ − 1). A high value in this index (near to 1) indicates that there exist well-defined selection criteria for the species regarding a specific illness category on behalf of the informants and/or that they are in full agreement in using that species for a specific use, while a low index (near to 0) indicates the choice of the species was random and that there is no consensus among the informants on the medicinal use of the species [46]. The result will be compared with others known to Portugal [41, 43].

## Results and discussion

### Diversity of medicinal plants and plant parts used

In Table 2, we list the plants cited by a minimum of three different informants using the criteria of Le Grand and Wondergem and Johns et al., cited in Bonet et al., [47], organized in alphabetical order by the corresponding botanical families (46). This table also contains other data such as categories and subcategories used, as well as methods of preparation and administration and voucher numbers.

The 10 botanical families with more *taxa* were *Rosaceae* (12 species), followed by *Asteraceae*, *Fabaceae* and *Lamiaceae* (eight species each), *Solanaceae* (seven), *Apiaceae* and *Poaceae* (four species each), *Brassicaceae*, and *Cucurbitaceae* and *Malvaceae* (three species each). The remaining 36 botanical families were represented by only one or two species. The most represented families coincide with those of other ethnobotanical studies in the Mediterranean area with this same methodology [41–43, 47–54].

These families predominate in local folk medicine, probably because they are widely represented in the local flora [47].

The 11 botanical families with more mentions were *Malvaceae* (504), *Lamiaceae* (220), *Rosaceae* (163), *Poaceae* (161), *Rutaceae* (151), *Asteraceae* (134), *Equisetaceae* (128), *Apiaceae* (100), *Amaryllidaceae* (97), *Oleaceae*, and *Solanaceae* (86 each). Note that botanical families with more *taxa*, mentioned above, are not necessarily the most cited.

**Fig. 2:**
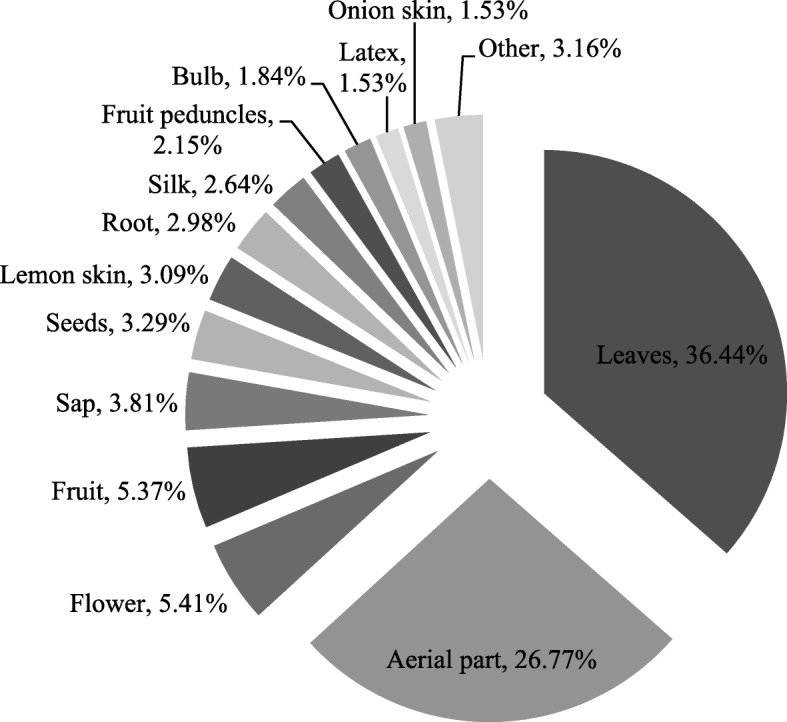
The plant parts used

As shown in Fig. 2, the plant parts used for medicinal proposes were in decreasing order: leaves, aerial part, flower, fruit, sap, seeds, lemon skin, root, silk, fruit peduncles, bulb, latex, and onion skin. The section “other” integrated the parts that were mentioned less than 1% (also in decreasing order: tuber, orange skin, young shoots, juice, resin, olive oil, pericarp, stem, corn kernel, mesocarp, petals, and seed coat).

The leaves, with the highest percentage of use, were also the parts most cited in other similar ethnobotanical works [42, 43, 47–49, 52, 54]. According to Bonet et al. [47], the easy accessibility of the leaves is the reason why they were used most of the times for medicinal purposes.

In most cases, the plant parts were used singularly and sometimes as a combination of two or more parts. For example, the aerial part and flower of *Chamaemelum nobile* (L.) All. were both used for diuretic purposes, or the aerial part, flower, and leaves of *Borago officinalis* L. were used to lower fevers.

### Preparation and administration mode of medicinal plants

**Fig. 3:**
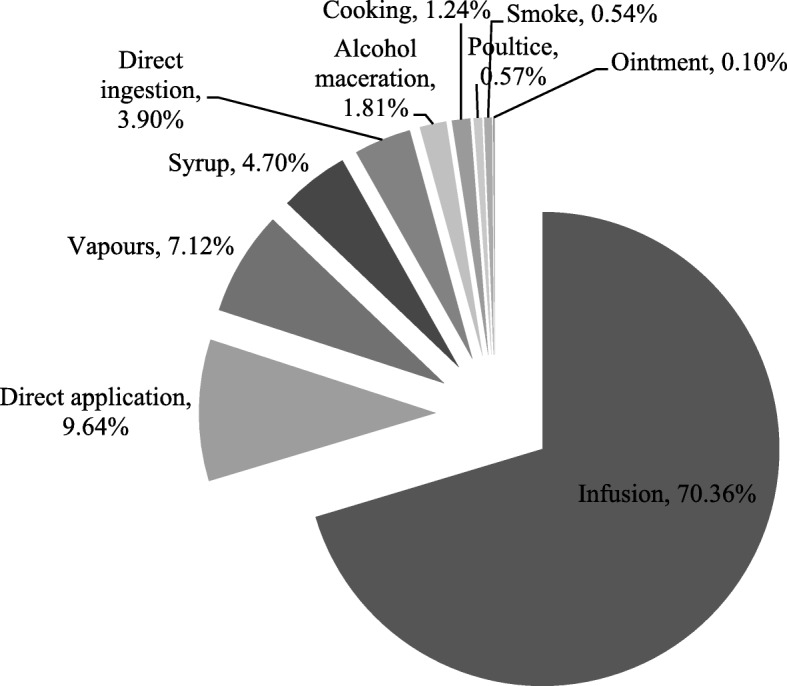
The preparation of medicinal plants

The preparation of medicinal plants is done in several ways, such as alcohol maceration, cooking, direct application, direct ingestion, infusion, ointment, poultice, smoke, syrup, and vapors. The most commonly used preparations were infusions (70% approximately), direct applications (10% approximately), and vapors (with 7% approximately). The remaining applications have about 13% of predominance (see Fig. 3). The prime method of preparation was the infusion, which corroborates Bonet et al. [47].

It is also important to point out that in most cases, it is very difficult to separate the procedures of decoction and infusion [47], whereby we considered, in this paper, the second method, which is the main method of preparation for oral and external administration.

Also, we can see that water is the vehicle for almost all oral and external preparations and it was used in the preparation of area or used to wash some parts of the body.

It should be noted that poultices were applied fundamentally over a piece of tissue.

Syrup, obtained mainly by sugar maceration, and alcohol maceration, was mostly used for rheumatism (with *Allium sativum* L., *Tamus communis* L., and *Atropa belladonna* L.) or for respiratory purposes like the treatment of bronchitis with patches (with *Rosmarinus officinalis* L.).

The smoke preparation, with only *Hyoscyamus albus* L., was applied for earache and toothache.

Almost all of the *taxa* are used alone as very few mixes have been identified. For example, in the production of poultices, flour was used, and in the preparation of ointments, olive oil and elderberry were used.

**Fig. 4:**
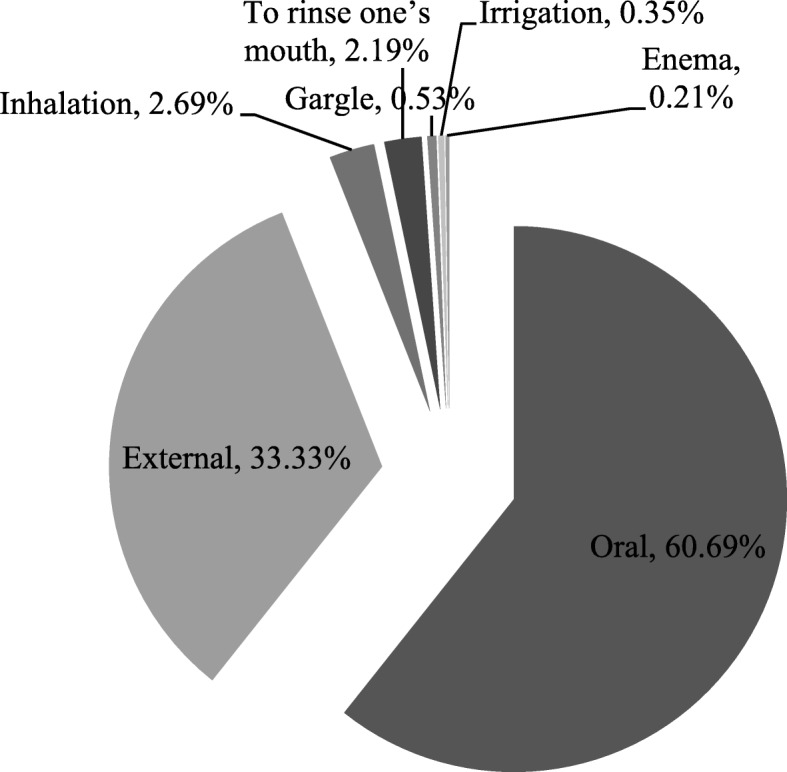
The administration processes

The Fig. 4 shows that the two main administration processes were oral (in 61% of cases, approximately) and external administration (in 33% of cases, approximately). In other situations were used inhalation, to rinse one’s mouth, gargle, irrigation and enema.

To conclude the general analysis of Table 2, it should be noted that only 13 *taxa* were used in a dried condition as the others were used in fresh and in fresh or dried. A few *taxa*, 19, were only used in the past, meaning they are no longer used by the populace even though the memory lingers as they were indeed mentioned in the interviews. The average number of plants referred per informant was approximately 26.68. The average number of use-reports referred per informant was equal to 36. The average number of use-reports referred per *taxon* is approximately equal to 26.74; the average number of different local Portuguese names per *taxon* was approximately equal to 2.9. Twenty plants were cited by 50% or more of interviewees.

### Local Portuguese plant names

Informants used 304 local names to refer to the 105 medicinal *taxa* cataloged. These names were checked against Portuguese publications that claim to contain all previously published common plant names [37–39]. We found several undocumented local names; for example, “bódanha”, “erva-da-infeção” and “erva-do-betadine”. It is also important to note that some local Portuguese names allude to their uses such as “quebra-pedra” (kidney stone of urinary category)—*Phyllanthus niruri* L.—or “erva-hepática” (liver of digestive category)—*Agrimonia eupatoria* L..

Table 2 has 315 vernacular names because some of them are repeated because different plants can have the same popular name (“pinheirinha”, “cavalinha”, “rabo-de-cavalo”, “tojo”, “hortelã”, “malva”, “malvas” and “limonete”).

### Diseases treated by medicinal plants

**Fig. 5:**
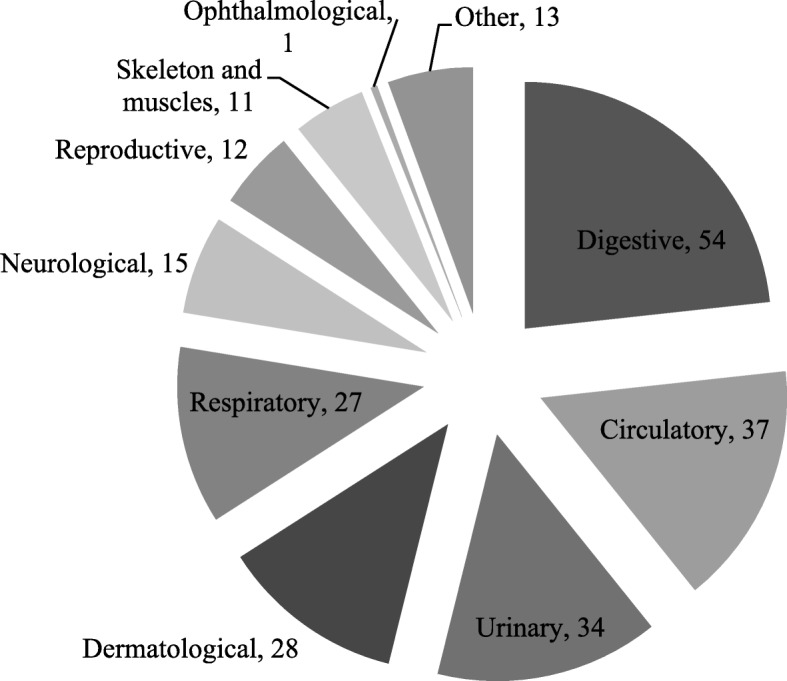
Number of the *taxa* for each illness category

The reported plants were grouped into 10 categories, based on the body systems, each of which is divided into several subcategories, based on the information gathered. Sometimes, the interviewees do not mention specific diseases or conditions; instead, they mention some organs (for example, liver or heart) or some processes (for example, cicatrizing or mineralizing). Figure 5 presents these 10 categories, with 54 *taxa* being included in the digestive category, 37 in circulatory category, 34 in urinary category, 28 in dermatological category, 27 in respiratory category, 15 in neurological category, 12 in reproductive category, 11 in the skeleton and muscle category, one in ophthalmological category, and 13 in other category (medicinal plants in contexts not covered in the previous categories). It is important to note that most plants are included in more than one category. The number of subcategories varied between two, in the ophthalmological category, and 22, in the dermatological category, a total of 95 subcategories. Several *taxa* appear in more than one category.

#### Digestive category

Fifty-four medicinal plants were reported for this group. The main species employed to treat digestive problems were *Hypericum perforatum* L., *Melissa officinalis* L., and *Geranium purpureum* Vill., with 70, 63, and 39 use-reports, respectively. In other works carried out in Portugal, these medicinal plants were also mentioned: *Hypericum perforatum* L. [41–43, 49, 54], *Melissa officinalis* L. [41–43, 49, 54], and *Geranium purpureum* Vill. [41, 42, 49].

#### Circulatory category

This is a prominent category of plant use, with 37 *taxa* and 14 subcategories used for purposes related to the circulatory system and blood. The species indicated with the highest number of use-reports were *Olea europaea* L. var. *europaea* (48), *Eriobotrya japonica* (Thunb.) Lindl. (25), and *Pterospartum tridentatum* (L.) Willk. (24). The first plant was referred in five studies [41–43, 49, 54] and the remaining in two [49, 54].

#### Urinary category

With 34 *taxa*, mainly used as an infusion, the most referred were *Zea mays* L. (71), also referred in other Portuguese studies [41–43, 49, 54]; *Prunus avium* (L.) L. (57), referred in three [42, 49, 54]; and the species belonging to the *Malvaceae* family (50 each) that were cited in two previous papers [42, 49]. It is curious to note that this is the only category for which fruit peduncles were used.

#### Dermatological category

The interviewees reported 28 plants to treat diseases related to this category. The administration method is fundamentally external. The *taxa* most cited were those belonging to the *Malvaceae* family (39 each), *Senecio serpens* G.D.Rowley (37), and *Aloe vera* (L.) Burm.f. (35). It has the largest number of subcategories (22) and the interviews reported that wounds can be treated by 19 different plants. Only the species of the *Malvaceae* family were referenced for similar purposes in Portugal [41, 42, 54].

#### Respiratory category

Twenty-seven medicinal plants were reported to be used in the treatment of respiratory problems, including *Eucalyptus globulus* Labill. (75), *Citrus limon* (L.) Osbeck (74), and *Daucus carota* subsp. *sativus* (Hoffm.) Schübl. & G. Martens (70). In other works carried out in Portugal, the first species was mentioned in four [41, 42, 49, 54] and the remainder in three, respectively [41, 42, 49] and [41, 42, 54].

#### Neurological category

Fifteen medicinal plants were considered beneficial in this category. The species with the highest number of use-reports were *Tilia cordata* Mill. (63), *Aloysia citrodora* Paláu (44), and *Melissa officinalis* L. (33). The same uses were referred in similar studies carried out in Portugal, namely [41, 42, 49, 54] for *Tilia cordata* Mill., [42, 49, 54] for *Aloysia citrodora* Paláu, and [42, 43, 49, 54] for *Melissa officinalis* L..

#### Reproductive category

The informants reported 12 *taxa*, which belong to nine botanical families (*Apiaceae*, *Cucurbitaceae*, *Equisetaceae*, *Fabaceae*, *Juglandaceae*, *Malvaceae*, *Poaceae*, *Rutaceae*, and *Urticaceae*). The species of *Malvaceae* family, with 38 use-reports, *Parietaria judaica* L. (14) and species from *Equisetaceae* family (12) were the most cited. The first family, *Malvaceae*, was referred in three studies [41, 42, 49] and the last, *Equisetaceae*, in two [49, 54] such as *Parietaria judaica* L. [49, 54].

#### Skeleton and muscles

Eleven *taxa* were mentioned. *Tamus communis* L., *Fraxinus angustifolia* Vahl, and *Allium sativum* L. were the species with the highest number of use-reports, 43, nine and seven, respectively. It is interesting to note that *Tamus communis* L. is only found in this group. These species were mentioned in works carried out in Portugal for the same uses, namely *Tamus communis* L. [54], *Fraxinus angustifolia* Vahl [41, 42, 49, 54], and *Allium sativum* L. [42, 54].

#### Ophthalmological category

It was reported one *taxon* in this group, *Rosa canina* L., with 36 use-reports. Note that this *taxon* is not referred to in any other category and petals were mentioned as the part used only in this instance. Carvalho [54] has also cited this *taxon* in association with this category.

#### Other category

This category has seven subcategories used in contexts unrelated or not connected with the previous categories (anti-cancerous, anti-inflammatory, aphrodisiac, earache, fever, mumps, and to slim). However, 13 of the plants that were reported in this category here were also mentioned in others.

Approximately 22.2% (10) of the botanical families were reported in relation to only one specific affliction, and approximately 44.8% (47) of *taxa* were reported in only one category.

### Quantitative assessment of ethnobotanical data

Characteristics such as homogeneity, importance, and cultural similarity were evaluated using quantitative indices which contributed to make solid comparisons with other independent Portuguese studies using the same methodology contrasting the results with previous works [41–43, 49, 54] as they relate to the traditional knowledge of medicinal plants used by the Montejunto population.

The ethnobotanical richness (*R*) is the number of *taxa* reported in each ethnobotanical study [40]. In this study, *R* is equal to 105. In similar studies carried out in Portugal, the values obtained were 88 [43], 104 [42], and 150 [41].

**Fig. 6:**
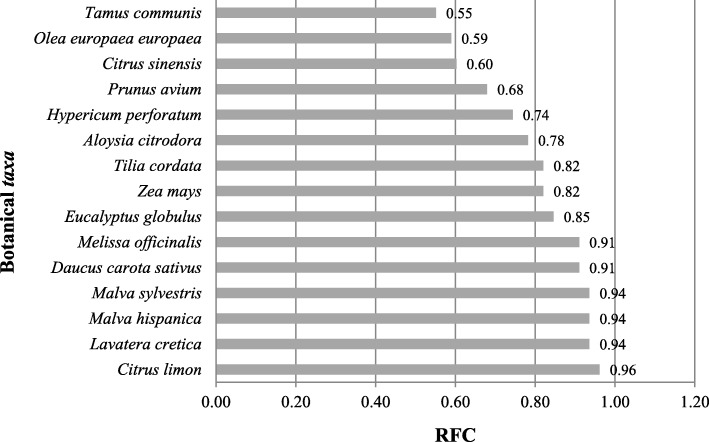
The botanical *taxa* with the highest RFC

As we can see in Table 2, the relative frequency of citation of the reported species ranges from 0.05 to 0.96. In Fig. 6, we have the 15 botanical *taxa* with the highest RFC, which reveals the importance of these species. The *Citrus limon* (L.) Osbeck has the highest value because it was mentioned by 75 informants, followed by *Lavatera cretica* L., *Malva hispanica* L., *Malva sylvestris* L., *Daucus carota* subsp. *sativus* (Hoffm.) Schübl. & G. Martens, and *Melissa officinalis* L.

**Fig. 7:**
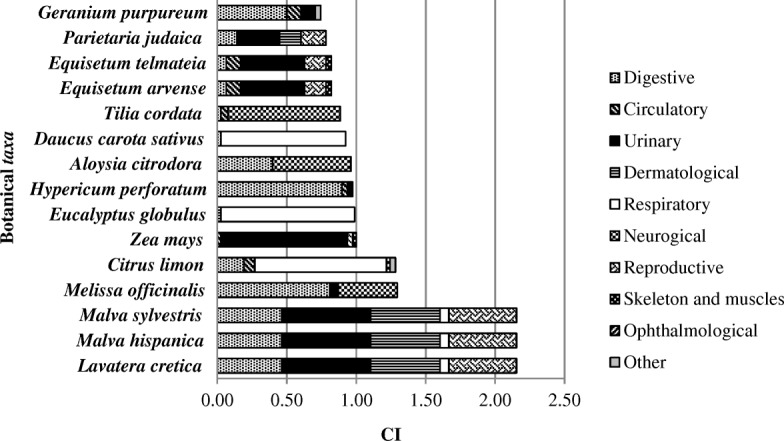
The botanical *taxa* with the highest CI

In Table 3 and Fig. 7, we present the data relating to the number of use-reports and the correspondent CI, for the botanical *taxa* with more use-reports. We can see that these 15 medicinal plants mentioned (14% of the total) correspond to approximately 50% of the total use-reports in Table 2. According to Table 2, CI ranges from 0.04, for *taxa* mentioned only by three informants and with only three use-reports (*Leucanthemum sylvaticum* (Brot.) Nyman, *Opuntia maxima* Mill., *Phaseolus vulgaris* L., *Ulex airensis* Esp.Santo, Cubas, Lousã, C.Pardo & J.C.Costa, *Ulex jussiaei* Webb, *Ulex minor* Roth, *Prunella vulgaris* L., *Oxalis pes-caprae* L., *Fumaria officinalis* L., *Phyllanthus niruri* L., *Hordeum vulgare* L. and *Valeriana officinalis* L.), to 2.15, for *Lavatera cretica* L., *Malva hispanica* L., and *Malva sylvestris* L. The low values of CI, according Tuttolomondo et al. [53], indicate that the local populations had little trust in some of the plants concerning the treatment of certain pathologies or as a strong indication of a gap or fading of traditional plant knowledge regarding their medicinal uses. Note that only three of the plants with CI equal to 0.04 are used nowadays (*Leucanthemum sylvaticum* (Brot.) Nyman, *Phaseolus vulgaris* L., and *Phyllanthus niruri* L.).

**Table 3:** The botanical *taxa* with more use-reports

*Taxa*	UR^a^	FC^b^	Number of different subcategories	CI^c^	Illness categories (in decreasing order)
*Lavatera cretica* L.	168	73	14	2.15	Urinary, dermatological, reproductive, digestive, and respiratory
*Malva hispanica* L.	168	73	14	2.15	Urinary, dermatological, reproductive, digestive, and respiratory
*Malva sylvestris* L.	168	73	14	2.15	Urinary, dermatological, reproductive, digestive, and respiratory
*Melissa officinalis* L.	101	71	8	1.29	Digestive, neurological, and urinary
*Citrus limon* (L.) Osbeck	100	75	11	1.28	Respiratory, digestive, circulatory, other, and neurological
*Zea mays* L.	78	64	8	1.00	Urinary, reproductive, circulatory, skeleton, and muscles
*Eucalyptus globulus* Labill.	77	66	6	0.99	Respiratory and dermatological
*Hypericum perforatum* L.	76	58	8	0.97	Digestive, urinary, and circulatory
*Aloysia citrodora* Paláu	75	61	4	0.96	Neurological and digestive
*Daucus carota* subsp. *sativus* (Hoffm.) Schübl. & G.Martens	72	71	3	0.92	Respiratory and digestive
*Tilia cordata* Mill.	69	64	4	0.88	Neurological, circulatory, and digestive
*Equisetum arvense* L.	64	40	15	0.82	Urinary, reproductive, circulatory, digestive, skeleton, and muscles
*Equisetum telmateia* Ehrh.	64	40	15	0.82	Urinary, reproductive, circulatory, digestive, skeleton, and muscles
*Parietaria judaica* L.	61	34	9	0.78	Urinary, reproductive, dermatological, and digestive
*Geranium purpureum* Vill.	58	39	10	0.74	Digestive, urinary, circulatory, and other

^a^The number of use-reports

^b^The number of informants that referred the *taxon*

^c^CI=UR/*N*

The average *F*_IC_ value for all categories is 0.90, higher than the value obtained in other Portuguese studies [41, 43], which are respectively 0.85 and 0.48. The high *F*_IC_ values found in most of the medicinal categories reflect a high level of homogeneity in consensus among the users and indicate that natural remedies are still considered extremely effective

In Table 4, we have presented some important data for each category, namely the number of *taxa*, the incidence, the number of use-reports, the *F*_IC_, and the medicinal importance. Through the analysis of this table, we find that *F*_IC_ varies from 0.69 for the category “other” to 1.00 for the ophthalmological category. Note that the value of 1.00 for the ophthalmological category is due to the fact that all informants indicated the same purpose for the *taxon* they mentioned.

**Table 4:** Informant consensus factor (*F*_IC_) and medicinal importance (MI) of medicinal plants

Illness category	Number of *taxa* (*n*_*t*_)^a^	Incidence (%)^b^	Number of use-reports (*n*_ur_)	Informant consensus factor (*F*_IC_)^c^	Medicinal importance (MI)^d^
Digestive	54	51.43	659	0.92	12.20
Circulatory	37	35.24	278	0.87	7.51
Urinary	34	32.38	483	0.93	14.21
Dermatological	28	26.67	427	0.94	15.25
Respiratory	27	25.71	375	0.93	13.89
Neurological	15	14.29	254	0.94	16.93
Reproductive	12	11.43	169	0.93	14.08
Skeleton and muscles	11	10.48	87	0.88	7.91
Ophthalmological	1	0.95	36	1.00	36.00
Other	13	12.38	40	0.69	3.08

^a^A *taxon* may be listed in several of the categories of medicinal usage

^b^As percentage of records on the total of 105 records

^*c*^*F*_IC_ = (*n*_ur_ *− n*_*t*_)/(*n*_ur_ − 1)

^d^MI = *n*_ur_/*n*_*t*_

### Comparison with similar studies from the Mediterranean region

In the following, taking into account the quantitative data collected, during the interviews, we present solid comparisons with other similar studies using the same methodology. In this context, Table 5 shows some quantitative data on medicinal plants in 11 regions, including this study. The data collected from various regions of Portugal, Spain, and other Mediterranean countries [41, 43, 48, 51–53, 55–58] are presented by the year of publication.

**Table 5:** Quantitative ethnobotanical data in several Mediterranean territories

Regions	*R* ^a^	*F* ^b^	*N* ^c^	*R*/*N*	*F*_IC_^d^
Montseny, Spain—2003 [55]	351	89	172	2.04	0.91
Serra de São Mamede, Portugal—2003 [41]	150	–	45	3.33	0.85
Western Pyrenees, Spain—2007 [56]	92	–	88	1.05	0.65
Trás-os-Montes, Portugal—2009 [43]	88	42	46	1.91	0.48
Alt Empordà, Spain—2009 [57]	335	80	178	1.88	0.91
Middle Navarra, Spain—2011 [51]	198	60	276	0.72	0.86
Eastern Mallorca, Balearic Islands—2012 [52]	121	64	42	2.88	0.71
Nebrodi Regional Park Sicily, Italy—2014 [53]	90	44	226	0.40	0.54–0.94
Bozyazı, Turkey—2015 [58]	159	55	178	0.89	0.11–0.74
Mount Hermon, Lebanon—2015 [48]	124	42	53	2.34	0.66–0.94
Serra de Montejunto, Portugal	105	46	78	1.35	0.90

^a^The ethnobotanical richness

^b^The number of families

^c^The number of informants

^d^*F*_IC_ = (*n*_ur_ − *n*_*t*_)/(*n*_ur_ − 1)

The table shows that in terms of ethnobotanical richness, *R*, there are three studies with lower values than this study. However, this corresponds to the second best value for Portugal. The value obtained for *F*_IC_, 0.90, is similar to the higher values recorded for the other studies, which indicates a high degree of consensus among the informants.

### Medicinal plants reported by one or two informants

The previous statistical study was based on the plants reported by three or more independent informants. However, it is also considered important to present the list of plants that were reported by only one or two informants (Table 6), because, although they may be less statistically significant, they may reflect the acculturation that has occurred in the last half-century in the industrialized western countries, such as those of Western Europe, where, at least partially, a modern culture is being adopted in detriment of the traditional one [59].

**Table 6:** Plants with medicinal uses reported by one or two informants

Botanical family, scientific name	Local Portuguese names	FC^a^	Popular use	Part(s) used	Preparation	Administration	Condition	Actual use	Voucher number
*Apocynaceae*									
*Vinca major* L.	Vinca, pervinca, pervinca-maior, congossa-maior	1	Circulatory—diabetes	Leaves	Infusion	Oral	Fresh	No	LISI 117/2019
*Asparagaceae*									
*Agave americana* L.	Piteira, piteira-de-boi, piteira-brava, pita	1	Respiratory—cough	Leaves	Syrup	Oral	Fresh	No	LISI 397/2019
*Urginea maritima* (L.) Baker	Cebola-albarrã, cebola-marítima	1	Dermatological—wounds	Stem	Direct application	External	Fresh	No	LISI 128/2019
*Asteraceae* (*Compositae*)									
*Helianthus annuus* L.	Girassol, helianto	2	Circulatory—cholesterol	Leaves	Infusion	Oral	Fresh	Yes	LISI 398/2019
*Crassulaceae*									
*Umbilicus rupestris* (Salisb.) Dandy	Umbigo-de-vénus, conchelos, caracóis-das-paredes, sobreirinho-dos-telhados, coucelos, conchilros	1	Dermatological—callus	Leaves	Direct application	External	Fresh	No	LISI 129/2019
*Fagaceae*									
*Castanea sativa* Mill.	Castanheiro, castanheiro-comum, castanho	1	Respiratory—cough, throat	Leaves	Infusion	Oral	Fresh or dried	Yes	LISI 118/2019
*Lamiaceae* (*Labiatae*)									
*Salvia officinalis* L.	Sálvia, salva, salva-comum, salva-das-boticas	2	Digestive—digestion	Leaves	Infusion	Oral	Fresh or	Yes	LISI 367/2019
			Reproductive—menopause	Leaves	Infusion	Oral	dried Fresh or dried	Yes	
*Lauraceae*									
*Laurus nobilis* L.	Loureiro, loureiro-comum, sempreverde, louro	1	Digestive – digestion	Leaves	Infusion	Oral	Fresh	Yes	LISI 119/2019
*Papaveraceae*									
*Papaver rhoeas* L.	Papoila, papoila-das-searas, papoila-vermelha, papoila-vulgar, papoila-ordinária	1	Neurological—tranquillizer	Flower	Infusion	Oral	Fresh	Yes	LISI 121/2019
*Passifloraceae*									
*Passiflora caerulea* L.	Flor-da-paixão, passiflora, martírios, maracujá-azul, cruz-de-cristo	1	Neurological—tranquillizer	Leaves	Infusion	Oral	Fresh	Yes	LISI 130/2019
*Plantaginaceae*									
*Digitalis purpurea* L.	Campainhas, dedaleira, flor-do-cuco, raposas, meias-do-cuco, erva-dedal, digital, abeloura	2	Circulatory—heart	Leaves	Infusion	Oral	Fresh	No	LISI 122/2019
			Urinary—kidneys	Leaves	Infusion	Oral	Fresh	No	
*Rosaceae*									
*Prunus spinosa* L.	Abrunheiro, abrunheiro-bravo, abrunho	1	Circulatory—heart	Leaves	Infusion	Oral	Fresh	No	LISI 123/2019
*Rutaceae*									
*Ruta chalepensis* L.	Arruda, arruda-dos-calcários, erva-das-bruxas, erva-da-graça	2	Digestive—appetite	Aerial part	Poultice	External	Fresh	No	LISI 124/2019
			Respiratory—asthma	Aerial part	Smoke	Inhalation	Fresh	No	
*Thymelaeaceae*									
*Daphne gnidium* L.	Trovisco, trovisco-fêmea, trovisqueiro	2	Dermatological—warts	Latex	Direct application	External	Fresh	Yes	LISI 126/2019
*Tropaeolaceae*									
*Tropaeolum majus L.*	Chagas, capuchinhas, mastruço-do-perú	2	Other—antibiotic	Flower, leaves, seeds	Infusion	Oral	Fresh	Yes	LISI 125/2019

^a^The number of informants that referred the *taxon*

## Conclusion

This work was a crystallization of the experience and a way to take another look at the ethnopharmacological knowledge unearthed and explored throughout the experience. The fieldwork also allowed the inventory of 105 *taxa* with medicinal properties used by the population from the Protected Landscape of “Serra de Montejunto” (Lisbon District, Portugal), where studies on the traditional uses of plants were nonexistent. The plants were distributed among 10 categories and 95 subcategories according to their uses where digestive category included the largest number of plant species.

The botanical families *Rosaceae*, *Asteraceae*, *Fabaceae*, and *Lamiaceae* were those with the greatest species representation, which can be explained by the predominance of these families in the Mediterranean flora and also because they include some common plants. Although the properties of these families are used in pharmacology, they were not necessarily the most cited.

The leaves and aerial part were most often used in the preparation of medicinal concoctions, followed by the flower and fruit. The infusion and direct application preparations were the most frequently used and oral administration largely predominated over another one. The plant was also most often used fresh.

Most plants referred in this study are still in use today. Only 17 are no longer used at the present time because habits have changed. For example, due to the availability of medicinal products in pharmacies, *Atropa belladonna* L., *Ecballium elaterium* (L.) A. Rich., *Gomphrena globosa* L., *Hyoscyamus albus* L., and *Valeriana officinalis* L. are no longer favored.

The informants reported 315 common names for the medicinal plants, 11 of which are repeated because different plants have the same local name.

In quantitative terms, by analyzing some ethnobotanical data, we obtained similar results to other studies carried out in the Mediterranean region.

Two of the most cited plants, *Senecio serpens* G.D.Rowley and *Aloe vera* (L.) Burm.f., are not referenced in other studies, yet they are used by the locals. A more detailed analysis should be done relating to these two plants correlating with its use and the predominance of their mention by the interviewees.

As shown by our analysis of data collected, both through field research and interviews, the use of medicinal plants based on folk knowledge is still very much common in the region studied and still transmitted through the generations. Some of the younger generations living in rural areas turn to the plant knowledge of their ancestors instead of looking for a pharmacy. However, it is still possible for it to disappear from memory which is why these studies, where the memory is preserved and transmitted in writing, properly cataloged and analyzed, are vital. They also may encourage others, younger and/or outsiders, to take an interest in the plants and their uses as well as in investigating the traditions and the possibilities. However, a detailed analysis of each category and the benefits associated with the plants mentioned is still needed.

## Data Availability

The authors already included all data in the manuscript collected during the field surveys.
